# Zinc Status in Febrile Seizure: A Case-Control Study

**Published:** 2013

**Authors:** Mohammad Reza SALEHIOMRAN, Masoumeh MAHZARI

**Affiliations:** 1 Department of Pediatric Neurology, Non-Communicable Pediatric Diseases Research Center, Babol University of Medical Sciences, Babol, Iran

**Keywords:** Seizures, Febrile, Zinc

## Abstract

**Objective:**

Febrile seizure is the most common type of seizure in children. Their incidence is 2-5%. There are different hypotheses about relationship between neurotransmitters and trace elements (such as zinc) and febrile seizure. Zinc, as a major element of some enzymes, plays an important role in the central nervous system (CNS) and can affect some inhibitory mechanisms of CNS. The aim of the present study was to determine whether there were any changes in serum zinc level in children with febrile seizure in comparison with febrile children without seizure.

**Materials & Methods:**

This case-control study was performed on 100 patients aged 6 months to 6 years. This study was conducted between January and August 2012, on 50 children with febrile seizures (case) and 50 febrile children without seizures (control), that were referred to Amirkola Children Hospital (a referral hospital in the north of Iran). Two groups were matched for age and sex. The serum zinc levels in the both groups were determined by atomic absorption spectrophotometry method.

**Results:**

The mean serum zinc level was 0.585±0.166 mg/L and 0.704±0.179 mg/L in the case group and the control group, respectively (p=0.001). The mean serum zinc level was significantly lower in the febrile seizure group compared to the control groups.

**Conclusion:**

Our findings revealed that serum zinc level was significantly lower in children with simple febrile seizure in comparison with febrile children without seizure.

It can emphasize the hypothesis that there is a relation between serum zinc level and febrile seizure in children.

## Introduction

Febrile seizure is the most common seizure in children (1). It occurs in children aged 6 month to 6 years (2, 3). Their incidence is 2-5% or 4.8/1,000 person-year (4).

A family history of febrile convulsion (FC), head injury, maternal smoking and alcohol consumption during pregnancy, features of the acute underlying diseases accompanying the FC, and temperature peak have been associated with febrile seizures (5,6). To date, pathophysiology of febrile seizure remains unknown, but genetic factors or electrolyte disturbance may have a role in its occurrence or recurrence (7, 8). Gammaaminobutyric acid (GABA) is an important inhibitory neurotransmitter. Zinc has a regulatory effect on glutamic acid decarboxylase and the synthesis of GABA (2, 7). 

Lee et al. reported that there is an association between serum zinc level and febrile seizure (9). In another study by Heydarian et al., it was reported that the serum level of zinc was significantly lower in children with simple febrile seizure compared to febrile children without seizure (10).

Garty et al. reported that there was no relation between CSF zinc level and febrile seizure (11). onsidering the above-mentioned findings, we decided to design a study to investigate the serum zinc level in patients with febrile seizures in comparison with febrile children without seizure.

## Material & Methods

In this case-control study, 50 children aged 6 months to 6 years with febrile seizure (case), and 50 children with fever without seizure (control) admitted to the Amirkola Children Hospital, between January and August 2012, were enrolled in this study. This study was approved by the Ethics Committee of Babol University of Medical Sciences.

A febrile convulsion (FC) was defined as a seizure occurring in a child with documented temperature of at least 37.8oC. One single generalized seizure in 24 hours of fever period with duration less than 15 min and without focal features was defined as a simple FC. Whereas seizures were defined as complex if they lasted more than 15 min, had focal features, or occurred more than once in 24 hours (12). Children with a history of seizure, being younger than 6 month or older than 6 years, having zinc intake, having a history of febrile seizure, electrolyte disorder, structural brain damage, failure to thrive, or acute meningitis were excluded from the study, and children with simple febrile seizure, 6 months to 6 years old, a single generalized seizure, one seizure attack during illness, seizure duration less than 15 min and normal growth, were enrolled in the study. An age, sex matched control group (50 children) were selected among hospitalized children with a febrile illness (such as upper or lower respiratory tract infections, gastroenteritis, or urinary tract infection) without seizure. One mL blood sample was taken from all children at the first 6 hours of admission. All blood samples were centrifuged and separated serum was stored at -8°C. 

Serum zinc levels were measured by atomic absorption spectrophotometery method. The normal serum zinc level was considered 0.8–1.2 mg/L and zinc level less than 0.3 mg/L was defined as zinc deficiency. Data were analyzed by t-test, χ2, ANOVA, and kolmogorov- Smirnov test using SPSS version 18. A p–value less than 0.05 was considered as significant. 

## Results

We studied 50 children (30 males, 20 females) with febrile seizure and a control group of 50 patients (30 male, 20 female). The mean ages of patients in the febrile seizure and control group were 25.5±14.62 months and 26.62±15.66 months, respectively. 

The most common cause of fever was respiratory infections. From these 100 children, 4 patients (4%) had zinc deficiency in febrile seizure groups (serum zinc level was<0.3 mg/L), and no deficiency was seen in control groups. 

There was no significant relation between the mean serum zinc level and sex, age, and mean body temperature.

**Fig 1:**
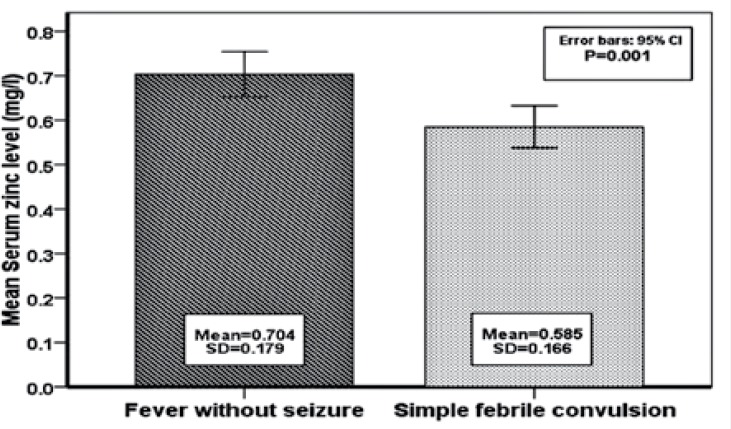
Mean of serum zinc level in fever without seizure and simple febrile convulsion groups

Table 1 and figure1 show that serum zinc levels were significantly lower in the febrile seizure group compared to the control group.

**Table1:** Serum Zinc Level in Febrile Convulsion and Fever Without Seizure Groups

	**Maximum**	**Minimum**	**Mean**	**SD**	**p-value**
Serum zinc level in febrile convulsion	1.015	0.245	0.585	0.166	0.001
Serum zinc level in fever without seizure	1.160	0.345	0.704	0.179	0.001

## Discussion

Our study showed that the mean serum zinc level were significantly lower in the febrile seizure group compared to the control group. Lee et al. in their study in Korea measured the serum zinc levels in 288 children with febrile seizure and 40 patients with afebrile seizure. Zinc levels in children with febrile seizure were significantly lower than those in children with afebrile seizure (9). 

Heydarian et al. reported that the serum level of zinc was significantly lower in children with simple febrile seizure compered to febrile children without seizure (10). In the Ehsanipour et al.’s study in Rasoul-e-Akram Hospital, serum zinc level was significantly lower in children with FC (cases group) compared to controls (children with having non-convulsive fever and children with non-febrile convulsion) (7). Ganesh et al. from India reported that serum zinc levels are lower in children with febrile seizure than in those with epileptic seizures and normal children (13). In accordance with other studies, the results of our study showed a relation between serum zinc levels and febrile seizure. On the other hand, in one report by Cho et al. from Korea in Pusan Hospital, there was no significant difference between serum zinc level of children with febrile seizure and that of control group. This difference with our result may be due to their small sample size (study was performed on 11 patients in each group) (14). Also, Garty et al.’s results do not support the hypothesis that febrile convulsions are related to reduce CSF zinc concentration. We think that this may be due to delayed CSF sampling after the febrile illness in their study (11). This finding of a variety of zinc related clinical disorders revealed the importance of zinc in human nutrition (15). Zinc is second to iron as the most abundant trace element in the body. More than 300 zinc metal enzymes occur in all six categories of enzyme system (15). Severe zinc deficiency is known to affect mental health, with varying degrees of confusion and depression being consistent with zinc enzymes have important role in brain development and function (15). Zinc can suppress some excitatory mechanisms in CNS. It can directly elevate the threshold of the seizure level by inhibiting N-methyl-D-aspartate (NMDA) receptors or through improving calcium inhibitory function (5, 7, 16, 17). In conclusion, our study results showed that children with febrile seizures had significantly lower serum zinc levels than those with fever without seizure. It is important to answer these questions that how zinc level plays role in the pathophysiology of febrile seizure and whether zinc supplementation could be effective in preventing febrile seizures. More and larger studies are required to answer these questions and also comparative study between serum and CSF zinc levels can be somewhat helpful.
